# Simulated performances of pixelated CsI(Tl) scintillation screens with different micro-column shapes and array structures in X-ray imaging

**DOI:** 10.1038/s41598-018-34852-3

**Published:** 2018-11-14

**Authors:** Hui Chen, Mu Gu, Xiaolin Liu, Juannan Zhang, Bo Liu, Shiming Huang, Chen Ni

**Affiliations:** Shanghai Key Laboratory of Special Artificial Microstructure Materials and Technology, School of Physics Science and Engineering, Tongji University, Shanghai, 200092 P. R. China

## Abstract

The performances of the pixelated CsI(Tl) scintillation screens based on oxidized silicon micro-pore array templates with different CsI(Tl) micro-column shapes and array structures in X-ray imaging were simulated using the Geant4 Monte Carlo simulation code. The shapes of the micro-columns include square, hexagonal and circular, and the array structures include square and hexagonal arrangements. The pitch size of the pixelated CsI(Tl) scintillation screens was set to 4 µm, and the incident X-ray energy was set to 20 keV. The ratios of the number of scintillation photons that propagate along the CsI(Tl) micro-columns to the total number of scintillation photons of the micro-columns gradually decrease with the increase in total reflection time on the lateral surfaces of the micro-columns. However, these ratios are closely related to the shapes of the micro-columns and the incident positions of X-ray on the cross-sections of the micro-columns, especially for the circular micro-column. The sequence of bottom light outputs stimulated by a uniform flood field of X-ray from high to low corresponds to the circular, square and hexagonal CsI(Tl) micro-columns with the same cross-section areas. In addition, all spatial resolutions in terms of modulation transfer functions (MTFs) for the pixelated CsI(Tl) scintillation screens with square and hexagonal array structures are over 100 lp/mm. However, the resolution for the pixelated screen with the hexagonal array structure is approximately 8.5% higher than that for the screen with the square array structure. Moreover, the former screen has a higher detective quantum efficiency (DQE) than the latter screen at the same thickness. The pixelated CsI(Tl) scintillation screen with circular micro-column and hexagonal array structure in X-ray imaging has superior performance compared to other pixelated screens in this work.

## Introduction

CsI(Tl) scintillation screens have been widely used in X-ray imaging such as mammography, micro-tomography, and nondestructive testing^1,2^. With the development of X-ray imaging, the screens must have better spatial resolution and detection efficiency. To ensure adequate detection efficiency, the scintillation screens typically require a sufficient thickness^3^. However, increasing the thickness of the scintillation screens often comes at the expense of their spatial resolution because of the lateral spreading of scintillation light^4^. To overcome this limitation, CsI(Tl) scintillation screens with a needle-like columnar structure were developed through a thermal evaporation method^5^. The vertical columnar structure can suppress the lateral spreading of scintillation light and preserve the spatial resolution of X-ray imaging. However, this kind of structure is not perfect. The cross-talk of scintillation light between adjacent columns remains due to the lack of effective optical isolation. To solve this problem, the pixelated CsI(Tl) scintillation screens based on silicon micro-pore array templates were investigated^6^. The screens were made of silicon micro-pore array templates with oxidized reflective pore walls and filled with a CsI(Tl) scintillator^7^. Due to the total reflection, the scintillation light with an incident angle smaller than the critical angle α_c_ will be guided to propagate along the CsI(Tl) column, and the light that enters the pore walls will be basically absorbed by the pore walls and hardly transmit into the adjacent columns.

**Figure 1:**
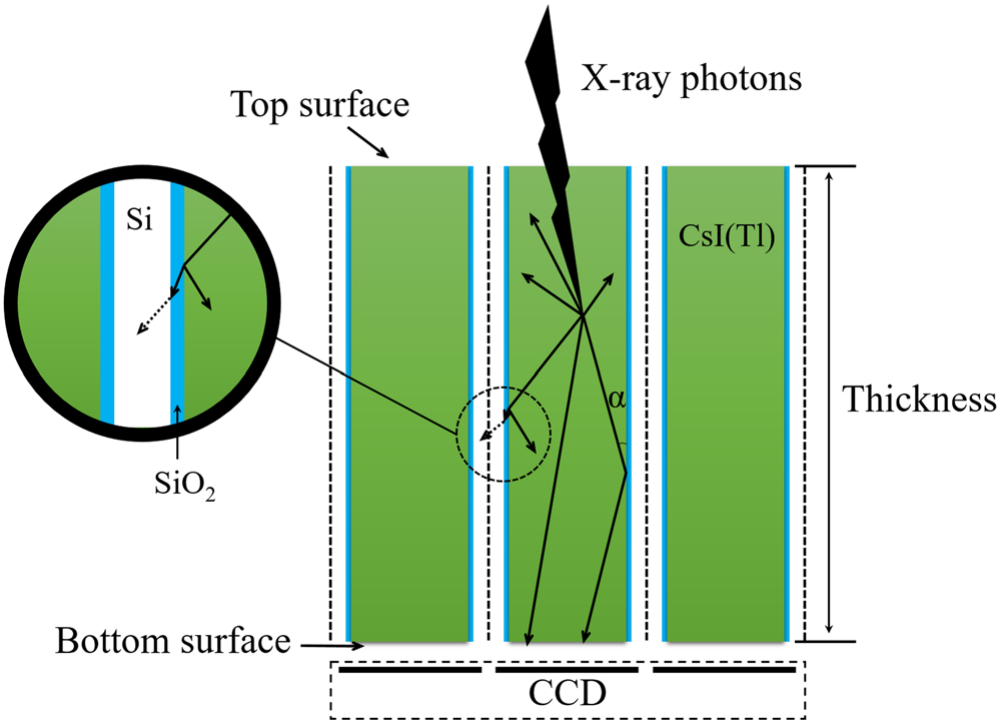
Principle of scintillation light of CsI(Tl) guided by silicon pore walls covered with SiO_2_ reflective layers.

The principle of the scintillation light guided by the pore walls with SiO_2_ reflective layers is illustrated in Fig. 1. For the shapes of the pores in the silicon micro-pore array templates used for scintillation screens, some are square columns^7–14^, some are hexagonal columns^11,12^, and some are circular columns^15^. However, there remains a lack of research on which type of column is more favourable to guide the scintillation light propagating along the column. In addition, the structure of the prepared pixelated CsI(Tl) scintillation screens is mostly a square array^7–14^. When the pitch size of the structured scintillation screen approaches the pixel size of the corresponding photoelectric device such as a CCD (charge coupled device) and CMOS (complementary metal oxide semiconductor), the Moiré patterns easily occur because of the interaction between the periodic scintillation array of the CsI(Tl) screen and the pixelated photoelectric device. To avoid this effect, the pixelated CsI(Tl) screens with a hexagonal array structure were developed^11,12^. The effect of the array structure of the scintillation screen on the spatial resolution and detective quantum efficiency (DQE) of X-ray imaging is also worth studying.

Geant4 is a Monte Carlo software toolkit for the simulation of the passage of particles through matter. It is used in many experiments and projects in various application domains, including particle physics, astrophysics, space science, medical physics and radiation protection^16^. Its functionality and modelling capabilities in event category, geometry module, tracking category, electromagnetic physics, and particle-matter interactions^17^ make it particularly suitable for simulating the behaviour of scintillation screens in X-ray imaging. In this work, the guide effects of the oxidized silicon micro-pores filled with a CsI(Tl) in different shapes on scintillation light were simulated by using Geant4. The performances of the pixelated CsI(Tl) scintillation screens with different array structures in X-ray imaging were also simulated and compared.

## Simulation Method

### Screen construction

The silicon micro-pore array templates with different pore shapes and array structures can be fabricated by photolithography and then by inductively coupled plasma (ICP), electrochemical etching, etc. A SiO_2_ layer on the inner wall of the silicon pore can be grown by the thermal oxidation method. Because the refractive index of SiO_2_ (n = 1.46) is less than that of CsI(Tl) (n = 1.74), the SiO_2_ layer can help to improve the guide effect of silicon pore on scintillation light. The pixelated scintillation screens are made of oxidized silicon micro-pore array templates filled with a CsI(Tl) scintillator. In this simulation, the pitch size of the pixelated CsI(Tl) scintillation screens was set to 4 μm (a relatively small pitch size that can be experimentally achieved)^12^, and the pore wall was set to consist of an 800 nm thick silicon layer covered with a 100 nm thick SiO_2_ layer at each side. The incident X-ray energy was set to 20 keV. The level of the Entrance Surface Air Kerma (ESAK) was set to 1.0 mGy, which could ensure the stability of the results.

**Figure 2:**
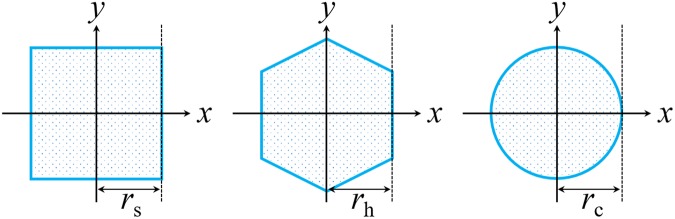
Cross sections of CsI(Tl) micro-columns in oxidized silicon micro-pores in different shapes. (left: square, middle: hexagonal, right: circular).

The light guide effects of CsI(Tl) micro-columns in oxidized silicon micro-pores with square, hexagonal and circular shapes and lengths of 10–100 µm were simulated. To compare the results of the CsI(Tl) micro-columns in these three shapes, their cross-sectional areas should be the same, i.e., $4{r}_{{\rm{s}}}^{2}=2\sqrt{3}{r}_{{\rm{h}}}^{2}={\rm{\pi }}{r}_{{\rm{c}}}^{2}$, where *r*_s_, *r*_h_ and *r*_c_ are the apothems of the square, hexagonal and circular micro-columns as shown in Fig. 2. When *r*_c_ was set to 1.5 µm, *r*_s_ and *r*_h_ were 1.33 and 1.43 µm, respectively.

**Figure 3:**
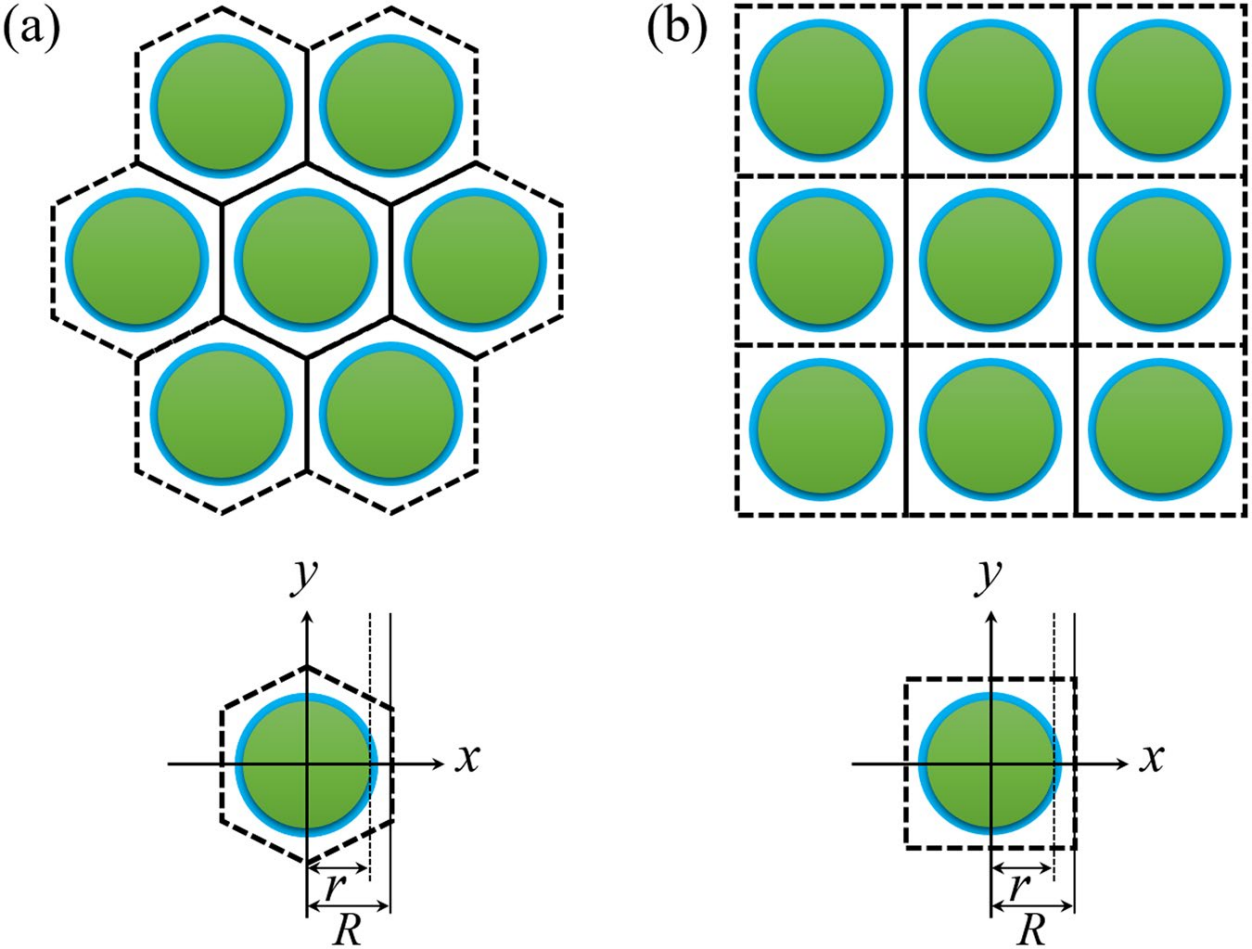
Pixelated CsI(Tl) scintillation screens with (**a**) square and (**b**) hexagonal array structures.

The pixelated CsI(Tl) scintillation screens with square and hexagonal array structures used in the simulation are shown in Fig. 3. The apothems of the circular CsI(Tl) micro-column and pixel were set to 1.5 µm (r) and 2.0 µm (R), respectively.

### Simulation process

The performances of the pixelated CsI(Tl) scintillation screens are characterized by the light output (LO), modulation transfer function (MTF) and detective quantum efficiency (DQE) of the detection system in X-ray imaging.

The mean number of generated scintillation photons per incident X-ray photon of pixelated screen was simulated by exposing the screen to a uniform flood field of X-ray. The scintillation photons can uniformly emit in all directions. However, the micro-column array structures can effectively guide the scintillation light that propagate along the columns. The photons that reach the backside of the screens were counted by an ideal CCD with a pixel size smaller than the pitch size of the pixelated CsI(Tl) scintillation screens. The refractive index of silicone oil for optical coupling between the screens and the CCD was set to 1.43^18^. The bottom light output was defined by the mean number of scintillation photons per incident X-ray photon detected by the CCD.

The spatial resolution of an X-ray imaging system is commonly described by its MTF. In this work, the MTF was simulated according to the standard edge measurement method^18^. An edge spread function (ESF) was acquired by using an edge phantom, which was the response of the X-ray imaging system to an opaque object with a straight edge. The MTF was calculated by Fourier transform of a line spread function (LSF), which was derived from the differential of the ESF along the direction (assuming the direction of the x-axis) perpendicular to the straight edge^19^

1 $${\rm{LSF}}(x)=\frac{d\,{\rm{ESF}}(x)}{dx},$$

2 $$\text{MTF}(f)=|\frac{1}{\sqrt{2\pi }}{\int }_{-\infty }^{\infty }\text{LSF}(x){e}^{2\pi ifx}dx|.$$

The value of the MTF at zero frequency was normalized to unity. The spatial resolution of the X-ray imaging system was defined by the frequency where MTF = 0.1.

The DQE is the most crucial imaging characteristic and refers to the efficiency of a detector to convert incident X-ray energy into an image signal. It was simulated using the recently developed Fujita-Lubberts-Swank (FLS) method^20–22^, which considers the MTF, noise power spectrum (NPS) and Swank factor. The expression for the DQE is as follows

3 $$\text{DQE}(f)=\text{DQE}(0)\cdot \overline{\text{NPS}(0)}\frac{{{\rm{MTF}}}^{2}(f)}{\overline{\text{NPS}(f)}},$$

where DQE(0) is the value of DQE at zero frequency calculated by the following equation^23^

4 $${\rm{DQE}}(0)={{\rm{A}}}_{Q}{{\rm{A}}}_{S},$$

where A_Q_ is the quantum detection efficiency of a screen, which can be evaluated as the ratio of the number of X-rays that deposit their energy in the screen to the number of X-rays incident on the screen; A_S_ is the Swank Factor^24^. The NPS is the output noise power spectrum of an X-ray imaging system; it quantitatively describes the amount and frequency of the noise in an imaging system. Each normal incident X-ray photon that interacts with a scintillation screen produces a 2D point spread function (PSF). The NPS produced by each detected X-ray photon along the x-axis direction was computed by summing the 2D PSF in the y-axis direction and taking the square of the magnitude of its Fourier transform

5 $${\rm{NPS}}(f)={|DF{T}_{x}(\sum _{y}{\rm{PSF}}(x,y))|}^{2}.$$

$\overline{{\rm{NPS}}(f)}$ was obtained by averaging NPS(*f*) over all detected events

6 $$\overline{{\rm{NPS}}(f)}=\frac{1}{N}\sum _{1}^{N}{\rm{NPS}}(f)$$

Finally, it was normalized by its value at zero frequency, i.e., $\overline{{\rm{NPS}}(0)}$ = 1.

### Parameter setting

The Monte Carlo simulation was performed using the Geant4 version 10.0 toolkit^16,17^. A number of parameters need to be used in the simulation: light yield, emission spectrum, optical attenuation coefficient, refractive index, etc. The scintillation light yield of CsI(Tl) was set to 54 photons/keV^25^. The emission spectrum of CsI(Tl) was obtained from the results of ref.^26^. The optical attenuation coefficient was determined by the transmission spectrum of the CsI(Tl) crystal^27^. The refractive index of CsI(Tl) in the wavelength range from 300 to 900 nm was obtained from ref.^28^. Additionally, the refractive indices and optical attenuation coefficients of Si and SiO_2_ were obtained from refs^28–30^. The refractive index of air was set to 1.

## Results and Discussion

### Guide effect of a CsI(Tl) micro-column on scintillation light

**Figure 4:**
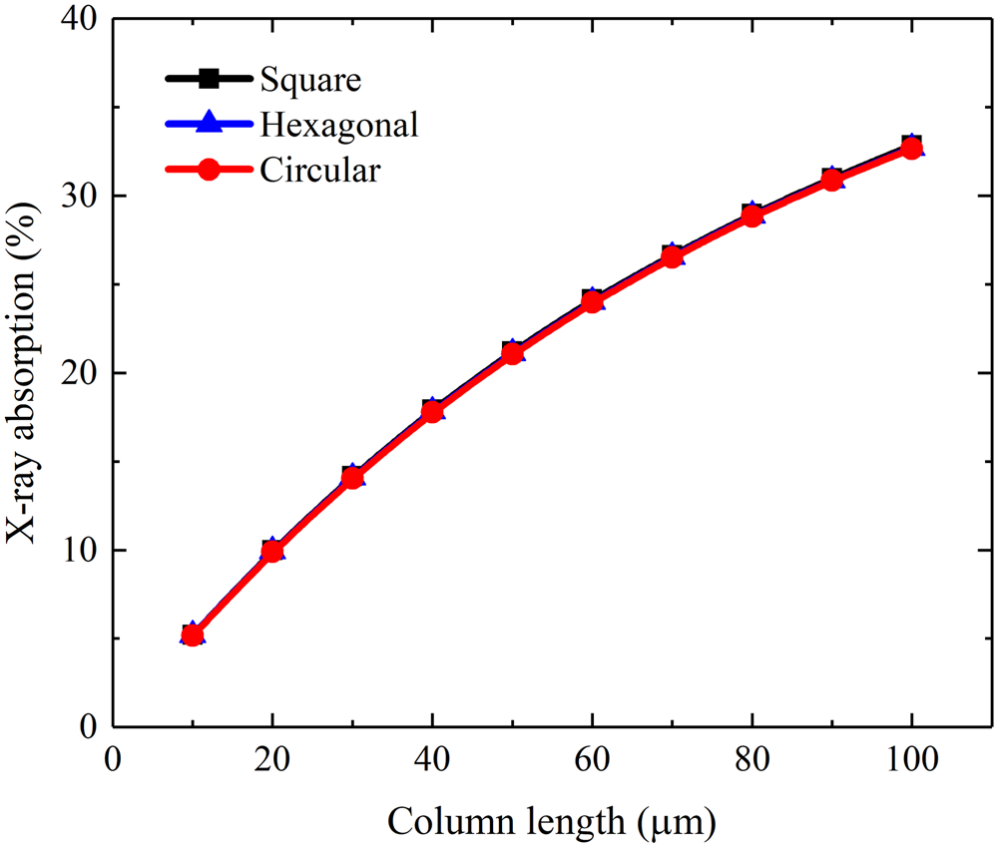
X-ray absorptions of different shapes of CsI(Tl) micro-columns as functions of their lengths.

**Figure 5:**
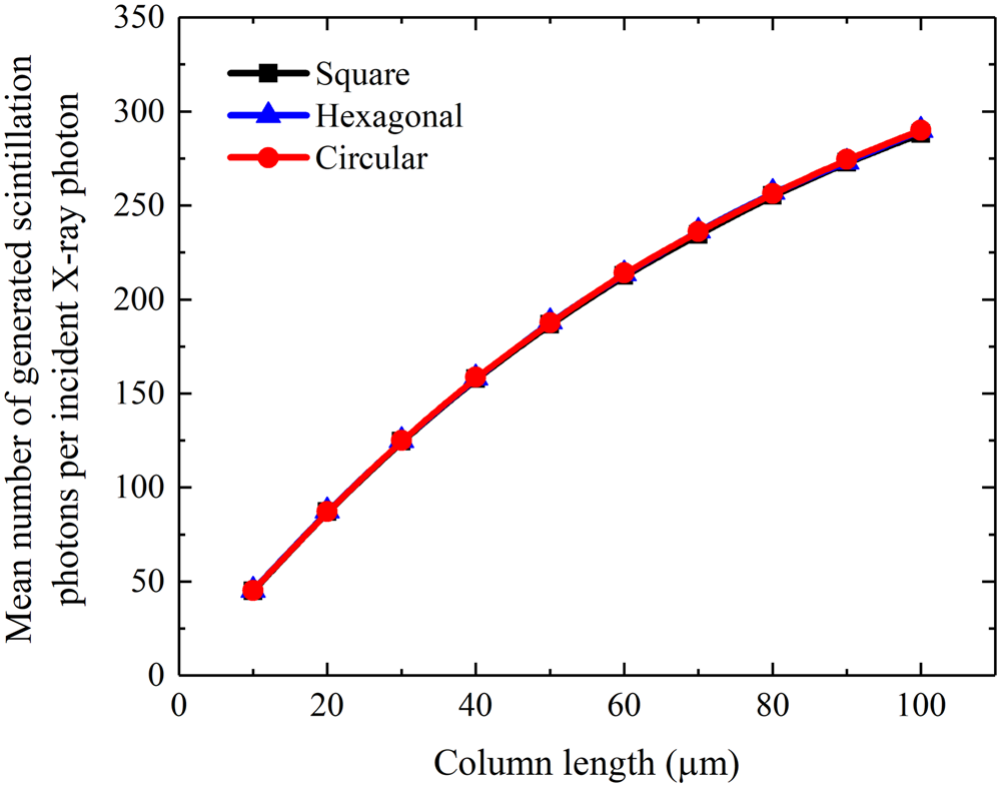
Mean number of generated scintillation photons per incident X-ray photon of different shapes of CsI(Tl) micro-columns as functions of their lengths.

**Figure 6:**
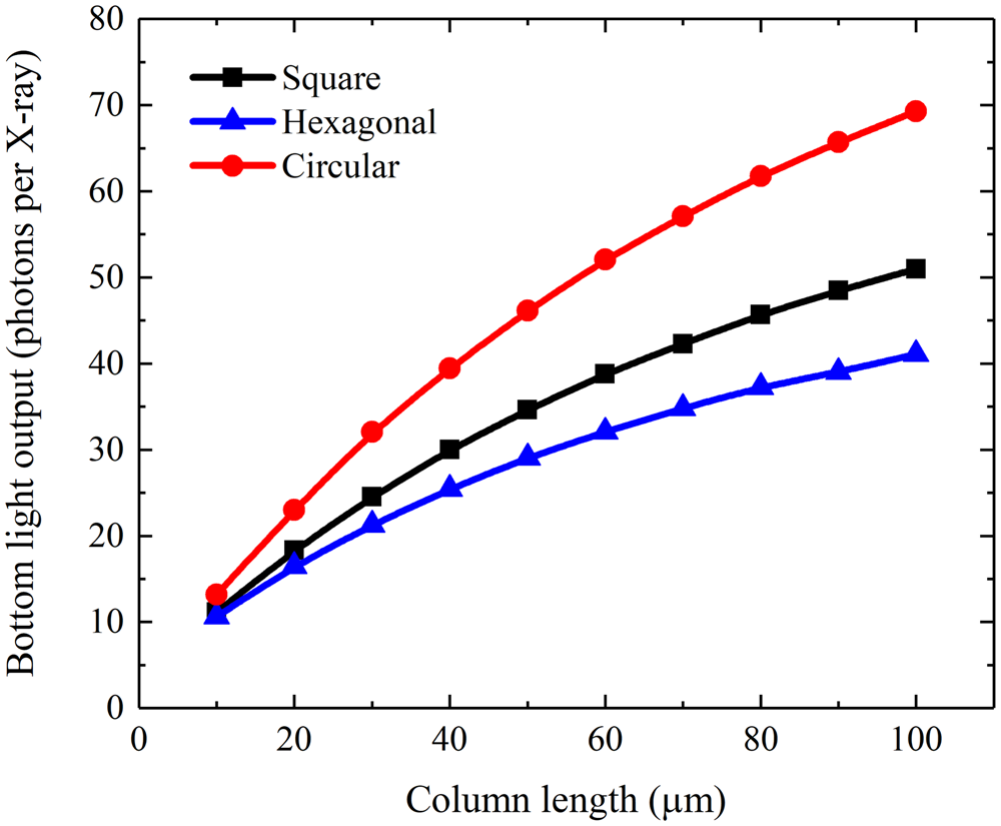
Bottom light outputs of different shapes of CsI(Tl) micro-columns as functions of their lengths.

**Figure 7:**
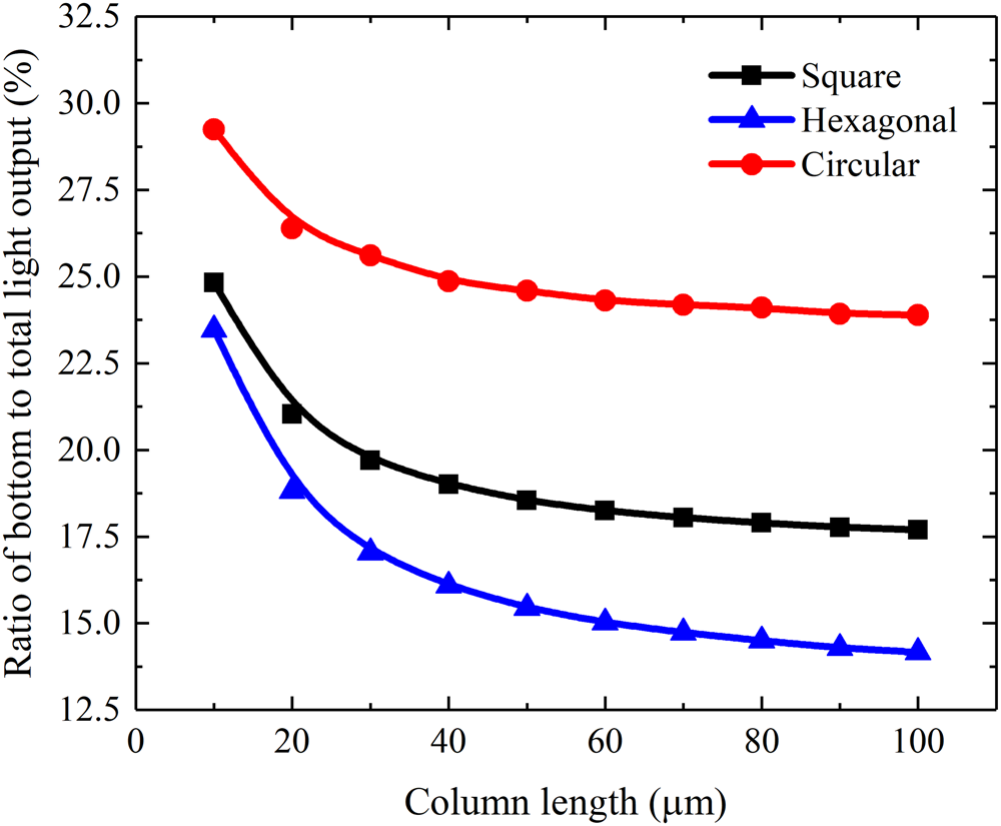
Ratios of the bottom light output to the mean number of generated scintillation photons of different shapes of CsI(Tl) micro-columns as functions of their lengths.

The simulated X-ray absorption and the mean number of generated scintillation photons per incident X-ray photon of a CsI(Tl) micro-column in the square, hexagonal or circular oxidized silicon micro-pore are shown in Figs 4 and 5. It can be seen that although they increase with the column length, as expected, they are independent of the micro-column shape because the micro-columns in three shapes have equal cross-sectional areas, i.e., 7.1 μm^2^. However, the bottom light output of the CsI(Tl) micro-column is closely related to its shape, as shown in Fig. 6. The bottom light output of the circular micro-column is approximately 1.4 and 1.7 times higher than the square and hexagonal ones, respectively, although they also increase with the column length. The results show that the guide effect of the circular CsI(Tl) micro-column on scintillation light is better

than that of the others. To better understand the light guide effects of the CsI(Tl) micro-columns in different shapes, the ratios of the bottom light output to the mean number of generated scintillation photons of the micro-columns in different shapes are shown in Fig. 7. They decrease at a decreasing rate with the increase in column length because of the increase in specific surface area of the micro-columns. Similarly, the ratio of the circular micro-column is better than that of the square one, and the ratio of the square micro-column is better than that of the hexagonal one.

**Figure 8:**
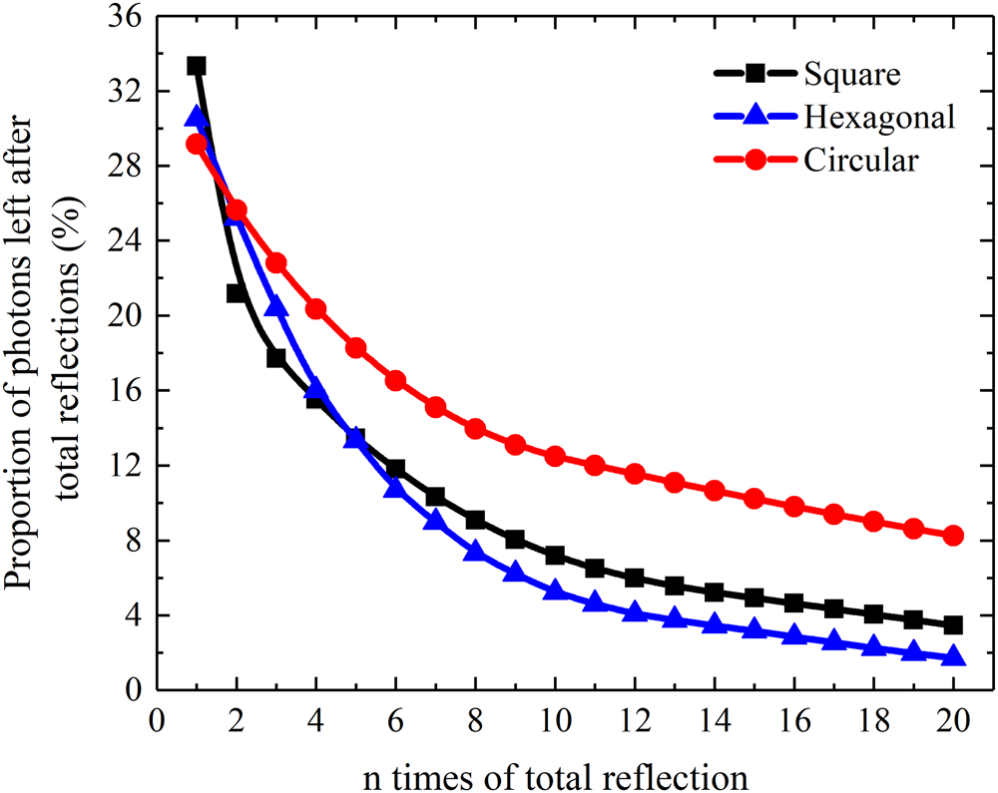
Proportions of the number of scintillation photons that pass through n total reflections to the total number of scintillation photons of CsI(Tl) micro-columns in different shapes.

To better understand the effect of the shape on the light guide behaviour of CsI(Tl) micro-column, the proportion of the number of scintillation photons that pass through n total reflections on the lateral surface of the micro-column to the total number of scintillation photons stimulated by the normal incident X-ray was simulated, as shown in Fig. 8. The order of the proportions after the first total reflection from high to low corresponds to the square, hexagonal and circular CsI(Tl) micro-columns. When the number of total reflections increases, the proportions gradually decrease because more scintillation photons escape from the columns. Meanwhile, the order of the proportions from high to low changes to circular, square and hexagonal CsI(Tl) micro-columns. Because most scintillation photons have to go through many total reflections to arrive at the bottom of the CsI(Tl) micro-columns, the circular micro-column has a better guide effect on scintillation light than the others.

**Figure 9:**
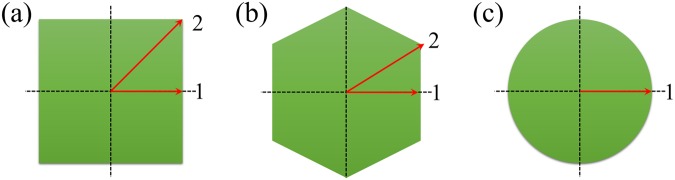
Schematic of the incident positions of X-rays on the cross sections of the CsI(Tl) micro-columns in three different shapes (positions on lines 1 and 2).

It was simulated that the proportion of the number of scintillation photons that pass through the total reflection to the total number of scintillation photons as a function of the incident position of X-ray on the cross section of the CsI(Tl) micro-column in different shapes. For simplicity, only those special positions on lines 1 (from the centre along the horizontal direction for all micro-column shapes) and 2 (from the centre along 45° and 30° with the horizontal direction for square and hexagonal micro-columns, respectively) were considered in the simulation, as shown in Fig. 9.

**Figure 10:**
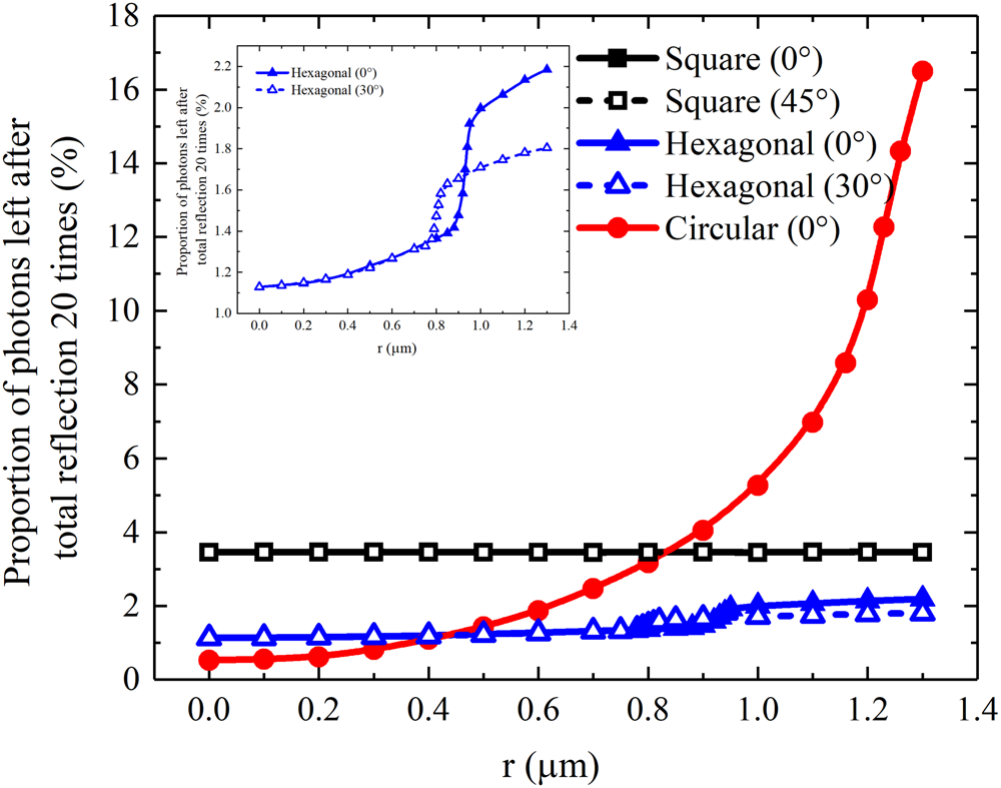
Proportions of the number of scintillation photons that pass through total reflection 20 times to the total number of scintillation photons as functions of the incident positions of X-rays on the cross sections of the micro-columns in three different shapes. r is a distance from the centre of the cross section to the incident position of the X-ray.

Figure 10 shows the proportions of the number of scintillation photons that pass through 20 total reflections to the total number of scintillation photons as functions of incident positions of X-rays on the cross sections of the micro-columns for three different shapes. When the incident positions of X-rays are at the centres of the cross sections of the micro-columns, the proportions for the micro-columns for all shapes are relatively limited (below 4%). The sequence of the proportions from high to low corresponds to the square, hexagonal and circular CsI(Tl) micro-columns. When the incident positions of X-rays depart from the centres of the cross sections, the proportions for the square and hexagonal micro-columns slightly vary, but the proportion for the circular micro-column exponentially increases. Thus, a circular micro-column has a better guide effect on scintillation light than the others.

### Performance of the pixelated CsI(Tl) scintillation screen

The performance of the pixelated CsI(Tl) scintillation screens with hexagonal and square array structures was simulated. Based on the above study, the circular CsI(Tl) micro-column has the best guide effect on scintillation light, so it was used to build the pixelated CsI(Tl) scintillation screens here.

**Figure 11:**
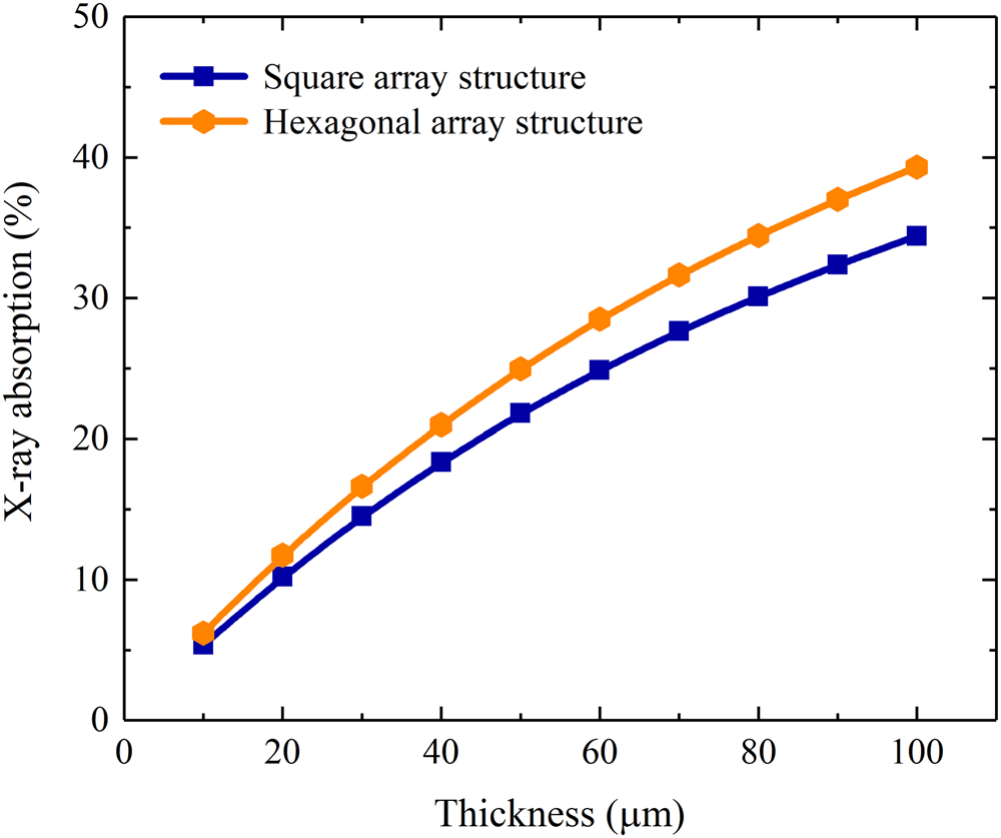
X-ray absorptions of the pixelated CsI(Tl) scintillation screens with different array structures as functions of their thicknesses.

**Figure 12:**
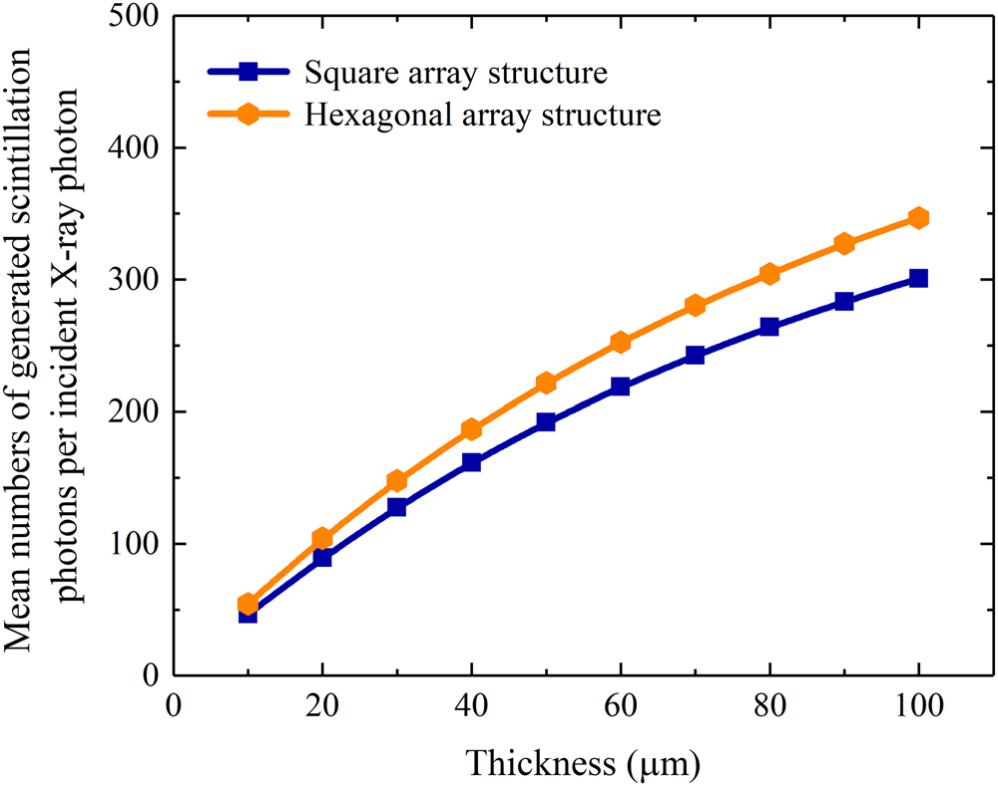
Mean numbers of generated scintillation photons per incident X-ray photon of the pixelated CsI(Tl) scintillation screens with different array structures as functions of their thicknesses.

The simulated X-ray absorptions and mean numbers of generated scintillation photons per incident X-ray photon of the pixelated CsI(Tl) scintillation screens with square and hexagonal array structures are shown in Figs 11 and 12. Although all of them increase with the screen thickness, the screen with the hexagonal array has an approximately 15% better result than the screen with the square array. The reason is that the arrangement of CsI(Tl) micro-columns in the hexagonal array is more compact. The fractions of CsI(Tl) areas of the screens with square and hexagonal array structures are approximately 44% and 51%, respectively.

**Figure 13:**
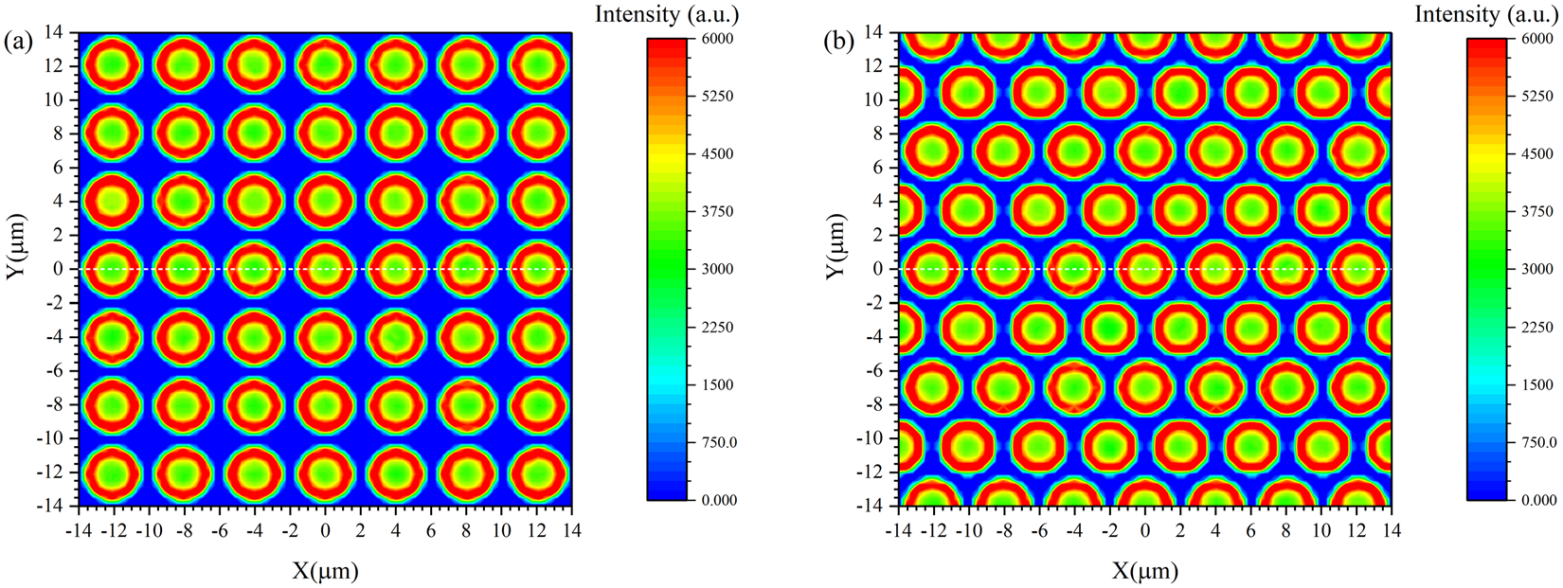
Distributions of scintillation light that reach the bottoms of the pixelated CsI(Tl) screens with (**a**) square and (**b**) hexagonal arrays.

The distributions of scintillation light that reach the bottoms of the pixelated CsI(Tl) scintillation screens with square and hexagonal array structures are shown in Fig. 13. The results reveal that the pixelated structures composed of oxidized silicon micro-pore array templates filled with a CsI(Tl) scintillator can not only effectively suppress the cross-talk of scintillation light among adjacent micro-columns but also guide the scintillation light to propagate along the direction of the micro-columns. The light intensity in the areas near the inside surfaces of the CsI(Tl) micro-columns is stronger than that in the other area. This observation is consistent with the result of Fig. 10, since the proportion of remaining scintillation photons after n total reflections increases exponentially when the generated positions of scintillation light depart from the cross-section centre of the circular micro-column. The percentage of the cross-talk on the bottom light output between adjacent micro-columns is less than 1%.

**Figure 14:**
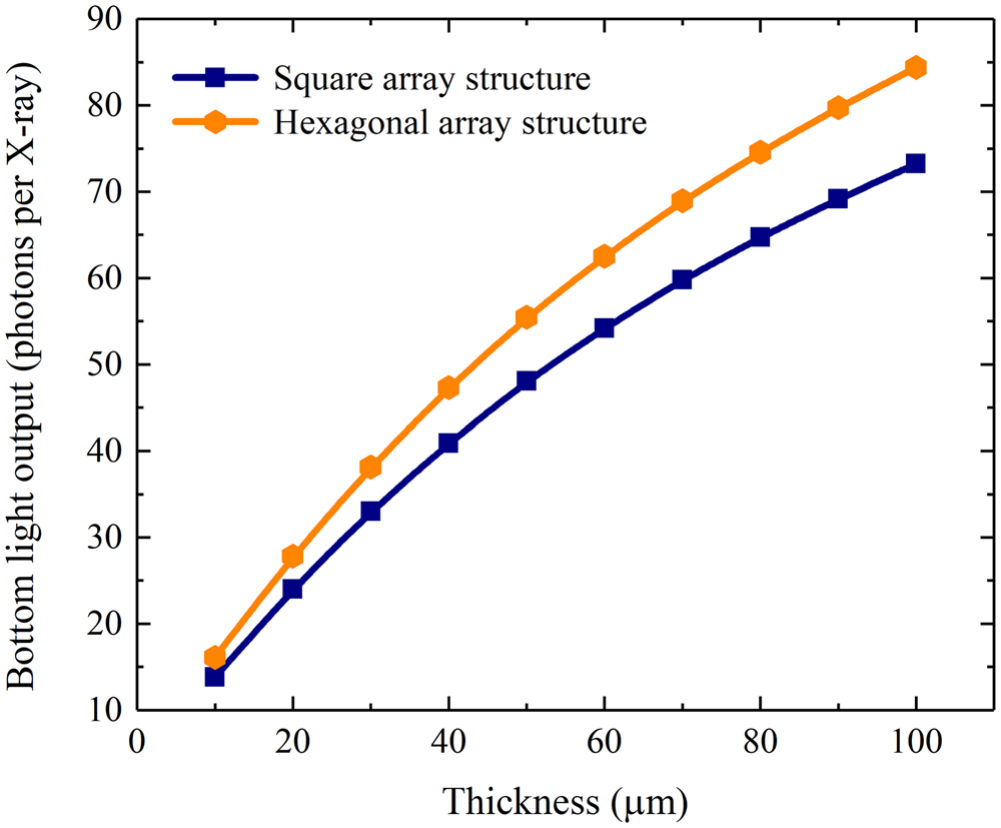
Bottom light outputs of the pixelated CsI(Tl) scintillation screens with different array structures as functions of their thicknesses.

**Figure 15:**
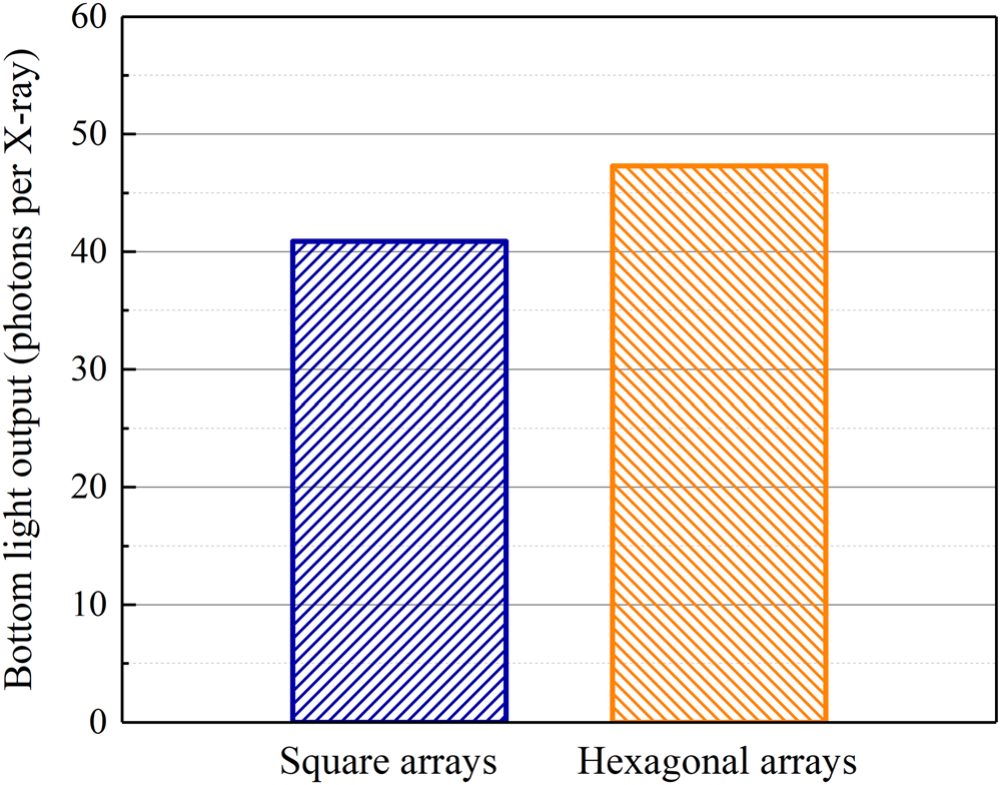
Bottom light outputs of the pixelated CsI(Tl) scintillation screens with the same 40 µm thickness but different array structures.

The bottom light outputs of the pixelated CsI(Tl) scintillation screens with square and hexagonal array structures are shown in Fig. 14. Similarly, they increase with the screen thickness; the bottom light output of the screen with the hexagonal array is higher than that of the screen with the square array. Figure 15 shows the bottom light outputs of both pixelated CsI(Tl) scintillation screens with the same 40 µm thickness but different array structures. The bottom light output of the screen with the hexagonal array is 15.7% higher than that of the screen with the square array.

**Figure 16:**
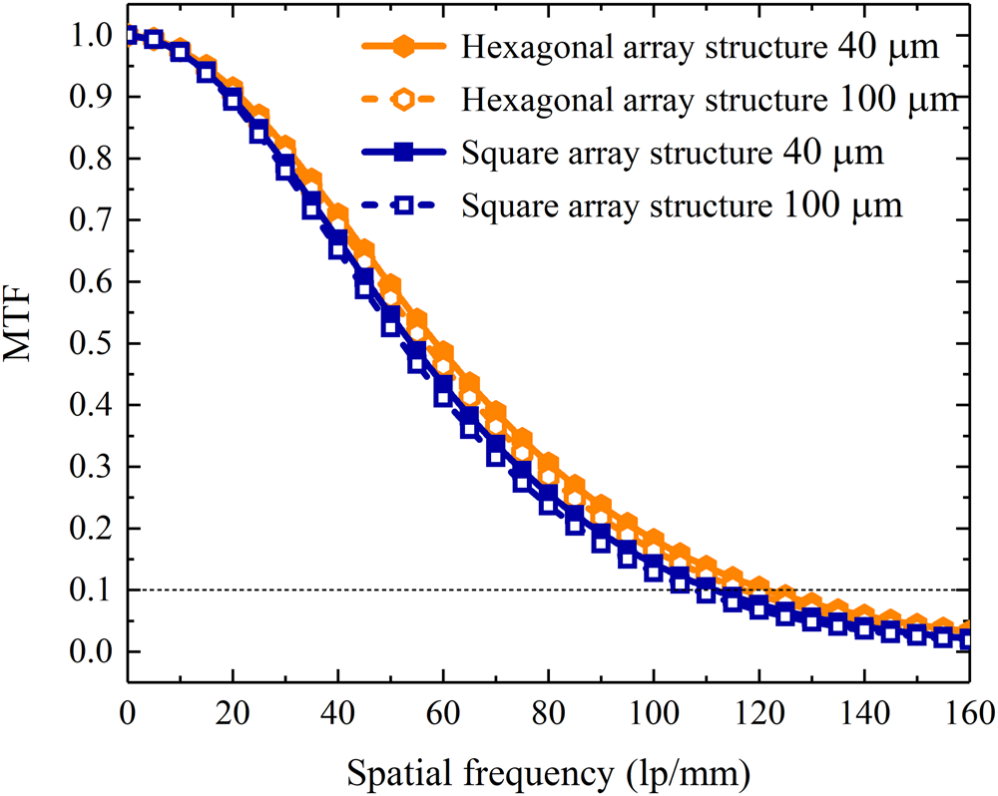
MTFs for the pixelated CsI(Tl) scintillation screens with square and hexagonal array structures at 40 μm and 100 μm thicknesses as functions of the spatial frequency.

The modulation transfer functions (MTFs) of the pixelated CsI(Tl) scintillation screens with square and hexagonal array structures at 40 μm and 100 μm thicknesses are shown in Fig. 16. The spatial resolution in terms of MTF for the screen is consistent with the experimental result^12,13^ of the pixelated CsI(Tl) scintillation screen with same pitch size, array structure and thickness. Furthermore, all spatial resolutions for both screens with square and hexagonal array structures slightly decrease with the increase in thickness. For the 40 μm and 100 μm thick square array screens, they are 111.5 and 107.8 lp/mm, respectively. However, for the 40 μm and 100 μm thick hexagonal array screens, they are 121.3 and 117.0 lp/mm, respectively. The spatial resolution for the pixelated CsI(Tl) scintillation screen with a hexagonal array structure is approximately 8.5% higher than that for the screen with a square array structure at the same thickness. The reason of the improvement is related to the compactness of the micro-column arrangement.

**Figure 17:**
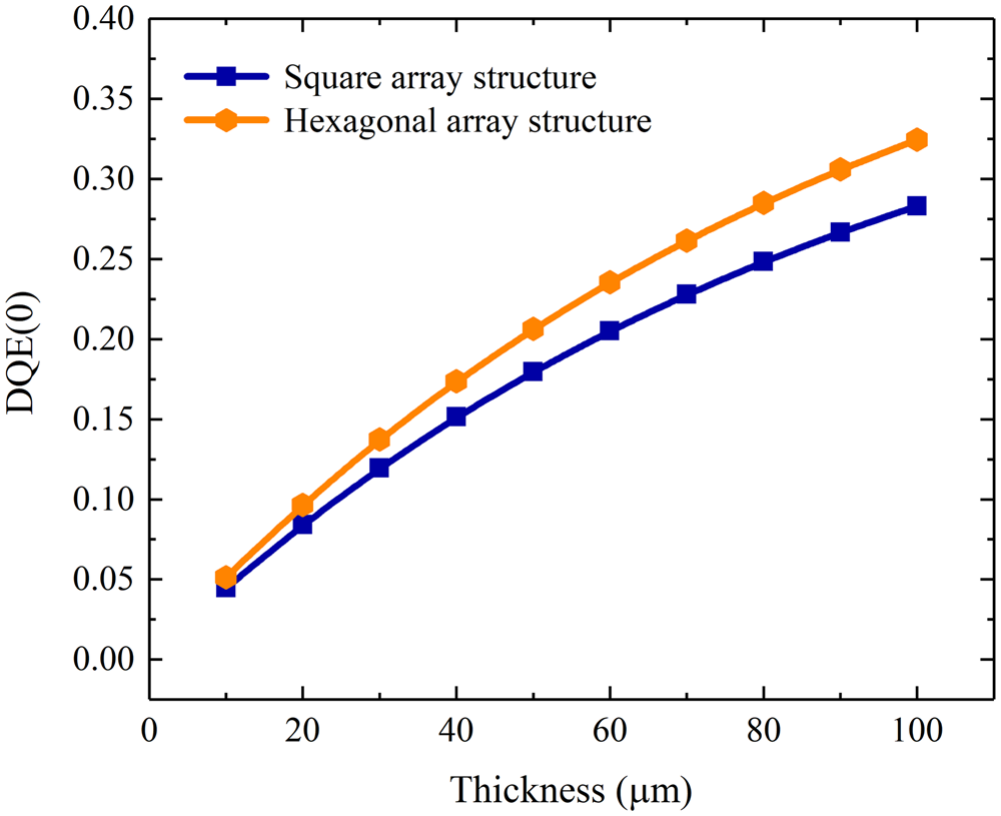
DQEs at zero frequency for the pixelated CsI(Tl) scintillation screens with square and hexagonal array structures as functions of the screen thickness.

**Figure 18:**
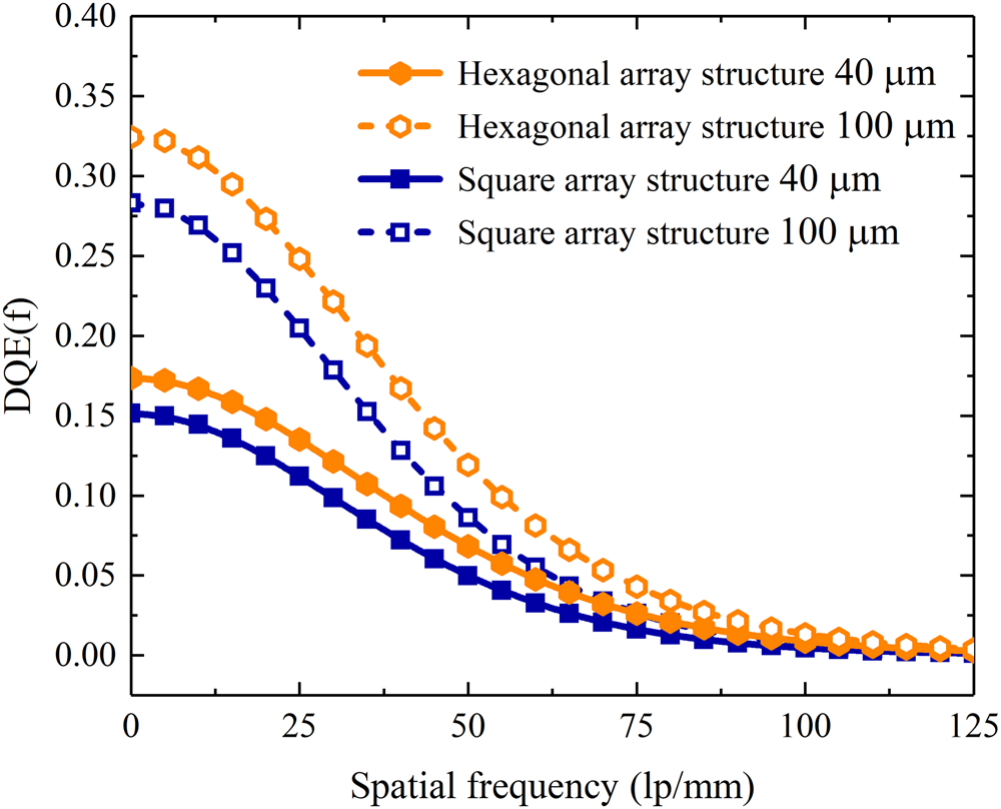
DQEs for the pixelated CsI(Tl) scintillation screens with square and hexagonal array structures at thicknesses of 40 μm and 100 μm as functions of the spatial frequency.

The detective quantum efficiencies (DQEs) at zero frequency for the pixelated CsI(Tl) scintillation screens with both square and hexagonal array structures as functions of the screen thickness are shown in Fig. 17. It can be seen that they obviously increase with the screen thickness, since the X-ray absorption simultaneously increases. Moreover, the DQE(0) for the pixelated screen with the hexagonal array structure is higher than that for the screen with the square array structure mainly because the filling rate of CsI(Tl) in the former screen is higher than that in the latter screen. To understand the overall variation of DQE with frequency, the DQEs for the pixelated CsI(Tl) scintillation screens with square and hexagonal array structures at thicknesses of 40 μm and 100 μm were simulated, as shown in Fig. 18. It can be seen that all DQEs decrease with the increase in spatial frequency because they are proportional to the square of the MTFs. Meanwhile, similar to DQE(0), DQE(f) for each of the structured screen increases with the screen thickness. Furthermore, the DQE(f) for the pixelated CsI(Tl) screen with the hexagonal array structure is higher than that for the screen with the square array structure at the same thickness, which implies that the former scintillation screen has better overall signal-to-noise transfer properties than the latter screen. The performance of the pixelated CsI(Tl) scintillation screen with the hexagonal array structure in X-ray imaging is superior to that of the screen with the square array structure.

## Conclusion

The X-ray imaging performances of the pixelated CsI(Tl) scintillation screens based on oxidized silicon micro-pore array templates with different CsI(Tl) micro-column shapes and array structures were simulated using the Geant4 Monte Carlo simulation code. The micro-columns had square, hexagonal and circular shapes, and the array structures included square and hexagonal arrangements. The guide effect of a filled CsI(Tl) micro-column in different shapes on the scintillation light was investigated. The proportions of the number of scintillation photons that propagate along the CsI(Tl) micro-columns to the total number of scintillation photons of the micro-columns gradually decrease with the increase in number of total reflections on the lateral surfaces of the micro-columns. However, these proportions are closely related to the shapes of the micro-columns and incident positions of X-ray on the cross sections of the micro-columns. When the incident positions of X-rays are at the centres of the cross sections of the micro-columns, the proportions for the micro-columns in all shapes are relatively limited. When the incident positions of X-rays depart from the centres of the cross sections, the proportions for the square and hexagonal micro-columns vary little, but the proportion for the circular micro-column increases exponentially. The bottom light output stimulated by a uniform flood field of X-ray of the circular micro-column is significantly higher than that in square and hexagonal columns with the same cross-section areas.

On the basis of the above research, the X-ray imaging performances of the pixelated CsI(Tl) scintillation screens with square and hexagonal array structures with a period of 4 μm were simulated, where the CsI(Tl) micro-columns are cylindrical. Although the bottom light outputs of the screens with both structures increase with the screen thickness, the result of the screen with the hexagonal array is approximately 15% higher than that of the screen with the square array. All spatial resolutions in terms of MTFs for both screens with square and hexagonal array structures exceed 100 lp/mm and only slight decrease with the increase in thicknesses. The resolution of the pixelated CsI(Tl) scintillation screen with the hexagonal array structure is approximately 8.5% higher than that of the screen with the square array structure at the same thickness. As expected, all DQEs for the pixelated CsI(Tl) scintillation screens with square and hexagonal array structures decrease with the spatial frequency and increase with the screen thickness. However, the DQE for the screen with the hexagonal array structure is higher than that for the screen with the square array structure at the same thickness, which implies that the former screen has a better overall signal-to-noise transfer properties than the latter screen. The performance of the pixelated CsI(Tl) scintillation screen with the circular micro-column and hexagonal array structure in X-ray imaging is superior to those of the other screens studied in this work.
